# Stem cell-based therapies for silicosis: mechanisms, sources, clinical translation, and emerging strategies

**DOI:** 10.3389/fimmu.2026.1745174

**Published:** 2026-02-18

**Authors:** Xinru Feng, Bo Xiao, Lixia Hou, Bingxi Zhang, Lincha Tian, Biwen Mo, Dong Yao

**Affiliations:** 1 Department of Respiratory and Critical Care Medicine, The Second Affiliated Hospital of Guilin Medical University, Guilin, China; 2 The key laboratory of Respiratory Diseases (Guilin Medical University), Education Department of Guangxi Zhuang Autonomous Region, Guilin, China; 3 The Laboratory of Respiratory Disease, The Affiliated Hospital of Guilin Medical University, Guilin, China; 4 Guangxi Clinical Research Center for Diabetes and Metabolic Diseases, Guangxi Health Commission Key Laboratory of Glucose and Lipid Metabolism Disorders, The Second Affiliated Hospital of Guilin Medical University, Guilin, China; 5 Guangxi Key Laboratory of Metabolic Reprogramming and Intelligent Medical Engineering for Chronic Diseases, Guilin Medical University, Guilin, China; 6 Scientific Research Center, Guilin Medical University, Guilin, China

**Keywords:** silicosis, stem cell therapy, pulmonary fibrosis, mesenchymal stem cells, clinical translation

## Abstract

Silicosis is an irreversible fibrotic interstitial lung disease triggered by chronic exposure to respirable crystalline silica (RCS). Currently, effective therapeutic interventions for this disease remain lacking, as existing clinical approaches are limited to mitigating disease progression rather than reversing or halting pathological changes. Stem cell-based therapies have emerged as a promising therapeutic modality for silicosis, leveraging their inherent biological properties to target key pathogenic cascades, such as NLRP3 inflammasome activation, TGF-β1/Smad-mediated fibrotic progression, and Th1/Th2 immune homeostasis imbalance. Notably, mesenchymal stem cells (MSCs) have advanced to early-phase (I/II) clinical trials for related pulmonary fibrotic diseases, demonstrating preliminary safety and potential for stabilizing lung function. This review synthesizes the latest advancements in stem cell-based therapeutic strategies for silicosis, with a systematic comparison of three key cell sources. The discussion encompasses adult stem cells, such as the readily accessible and immunomodulatory mesenchymal stem cells (MSCs) and the epithelium-regenerative airway basal stem cells (ABSCs), as well as the pluripotent but ethically debated induced pluripotent stem cells (iPSCs). Additionally, this review discusses critical challenges impeding the clinical translation of these therapies, including the standardization of GMP-compliant production processes, suboptimal homing efficiency of transplanted stem cells within the fibrotic pulmonary microenvironment, and inherent safety risks. Finally, this review highlights innovative translational strategies—such as CRISPR-engineered stem cells, stem cell-driven nano-delivery systems, and alveolar organoid models—and underscores the future potential of combination therapies and targeted approaches for silicosis-associated comorbidities. By integrating current knowledge, analyzing translational barriers, and exploring these forward-looking directions, this review aims to provide both theoretical insights and practical guidance for advancing the development and clinical application of stem cell-based therapies for silicosis.

## 1 Introduction

Silicosis is a prototypical occupational pneumoconiosis arising from chronic inhalational exposure to respirable crystalline silica (RCS), whose distinctive histopathological hallmarks encompass the progressive development of characteristic silicotic nodules and extensive fibrotic remodeling of pulmonary parenchymal. This pathological progression ultimately culminates in irreversible respiratory compromise (1). Despite clear understanding of its etiology, occupational exposure to hazardous concentrations of RCS persists as a global occupational health challenge, affecting millions of workers worldwide. Epidemiological surveillance indicates an annual incidence exceeding 20,000 new cases, leading the World Health Organization (WHO) to classify silicosis as a major contributor to the global burden of occupational diseases (2). Currently, clinical therapeutic measures for silicosis are limited to symptomatic treatment and terminal-stage pulmonary transplantation. Currently, clinical therapeutic options for silicosis are limited to symptomatic management and end-stage lung transplantation. While pharmacological interventions demonstrate constrained therapeutic efficacy and significant adverse effects, surgical approaches are substantially limited by donor organ scarcity, immunological rejection, and prohibitive costs. Consequently, developing novel treatment strategies that can precisely intervene in the molecular pathways of pulmonary fibrotic progression has become imperative.

Recent breakthroughs in regenerative medicine have unveiled the remarkable therapeutic potential of Stem Cells with their multipotent differentiation capacity, immunomodulatory properties, and paracrine signaling mechanisms emerging as pivotal elements in the management of refractory diseases (3–5). Stem cell-based therapies have emerged as a promising therapeutic approach for silicosis characterized by progressive pulmonary fibrogenesis. These interventions exert their therapeutic effects through comprehensive modulation of disease pathogenesis, including remodeling of the inflammatory pulmonary microenvironment, inhibition of fibrotic progression, and stimulation of alveolar epithelial regeneration and tissue repair (6–8). This review synthesizes contemporary progress in stem cell-based therapeutic interventions for silicosis, and focus on elucidating the molecular mechanisms underlying stem cell actions and evaluating their clinical translation potential across different cellular sources. Building upon this foundation, we analyze existing challenges in the field and explore the prospective applications of emerging biotechnologies such as gene-editing platforms and three-dimensional organoid models in stem cell therapy, aiming to provide theoretical insights and practical guidance for advancing both fundamental research and clinical translation of stem cell-based interventions for pulmonary fibrosis.

## 2 Pathological mechanisms

### 2.1 Deposition and phagocytosis of RCS

In the pathogenesis of silicosis, the deposition and phagocytosis of RCS constitute a critical initiating event. Owing to its structural similarity to pathogen-associated molecular patterns (PAMPs) or damage-associated molecular patterns (DAMPs), RCS is recognized by alveolar macrophages through multiple pattern recognition receptors (PRRs), including scavenger receptors (SRs), Toll-like receptors (TLRs), and nucleotide-binding oligomerization domain (NOD)-like receptors (NLRs). This recognition triggers receptor-mediated engulfment of silica particles, which in turn activates innate immune responses through nonspecific inflammatory pathways. Upon exposure to RCS, alveolar macrophages generate excessive reactive oxygen species (ROS), triggering lipid peroxidation of cell membranes and subsequent membrane damage. This membrane injury further compromises lysosomal integrity, resulting in NOD-like receptor family pyrin domain containing 3 (NLRP3) inflammasome activation and inducing a pro-inflammatory cascade. These pathological processes ultimately drive the development of pulmonary fibrosis, representing a fundamental pathological mechanism underlying silicosis pathogenesis (9, 10).

### 2.2 Inflammatory Cascade

In response to RCS stimulation, alveolar macrophages and epithelial cells activate both the NLRP3-ASC-caspase-1 inflammasome complex and NF-κB/MAPK signaling cascades. This dual activation mechanism leads to the production and release of pro-inflammatory cytokines and chemokines, initiating a sustained inflammatory cascade (7). These inflammatory mediators recruit diverse immune cells, particularly neutrophils and lymphocytes, to the injury site. Upon activation, neutrophils release myeloperoxidase (MPO) and matrix metalloproteinase-9 (MMP-9), which catalyze the production of ROS and reactive nitrogen species (RNS), thereby intensifying oxidative stress and potentiating tissue damage. Studies have confirmed that RCS exposure significantly upregulates the expression of Nicotinamide Adenine Dinucleotide Phosphate (NADPH) oxidase (NOX), which mediates the generation of superoxide anions (O_2_^−^) and hydrogen peroxide (H_2_O_2_) through the “respiratory burst” mechanism, ultimately establishing a vicious cycle of oxidative stress and mitochondrial dysfunction (10–12). Furthermore, activated neutrophils contribute to the degradation of alveolar basement membrane collagen and elastic fibers by releasing proteolytic enzymes such as MMP-9 and matrix metalloproteinase-12 (MMP-12), disrupting the alveolar epithelial-endothelial barrier and further aggravating the pathological progression of silicosis (13–15).

### 2.3 Fibrosis initiation and progression

Within the sustained inflammatory microenvironment, activated alveolar macrophages secrete transforming growth factor-β1 (TGF-β1) and platelet-derived growth factor (PDGF) through paracrine mechanisms. These cytokines synergistically drive the phenotypic transformation of pulmonary fibroblasts into activated fibroblasts. Specifically, TGF-β1 induces phosphorylation of the Smad2/3 signaling complex, facilitating its nuclear translocation. This transcriptional activation leads to marked upregulation of extracellular matrix (ECM) component synthesis, including type I collagen (COL-I) and fibronectin (FN). Concurrently, it suppresses the activity of collagenases, particularly matrix metalloproteinase-1 (MMP-1), resulting in impaired ECM degradation. This dual regulatory mechanism promotes pathological ECM accumulation, ultimately driving the progression of pulmonary fibrosis (16, 17). Furthermore, RCS directly binds to integrin receptors on alveolar epithelial cells, inducing epithelial-mesenchymal transition (EMT) through coordinated activation of the Wnt/β-catenin signaling pathway and Snail1-mediated epigenetic regulation. This transition confers migratory properties to epithelial cells while promoting the secretion of pro-fibrotic mediators, thereby accelerating pulmonary fibrotic progression (18, 19).

### 2.4 Immune response dysregulation

The progression of silicosis is closely associated with progressive dysregulation of Th1/Th2 immune homeostasis. During early-stage silicosis pathogenesis, the immune response exhibits a distinct Th1 polarization profile, characterized by upregulation of pro-inflammatory cytokines such as IFN-γ and TNF-α. These mediators drive macrophage activation and facilitate neutrophil infiltration, orchestrating a pro-inflammatory cascade that constitutes the characteristic inflammatory response in initial disease development. With disease progression, the immune response undergoes a gradual transition toward Th2 polarization, accompanied by elevated expression of fibrogenic cytokines, including IL-4 and IL-13. These mediators stimulate fibroblast proliferation and extracellular matrix deposition while promoting M2 macrophage polarization, collectively accelerating pulmonary fibrogenesis (20, 21). Concurrently, the depletion and functional impairment of circulating regulatory T cells (Tregs) compromise their immunomodulatory capacity, exacerbating dysregulated inflammation and fibrotic progression (22). Furthermore, RCS disrupts immune tolerance, triggering the generation of autoantibodies that form pathogenic immune complexes with pulmonary antigens. These immune complexes activate the complement system, inducing type III hypersensitivity reactions that inflict structural damage to the alveolar-capillary barrier and exacerbate pulmonary injury (23).

### 2.5 Oxidative stress, ferroptosis and DNA damage

**Figure 1:**
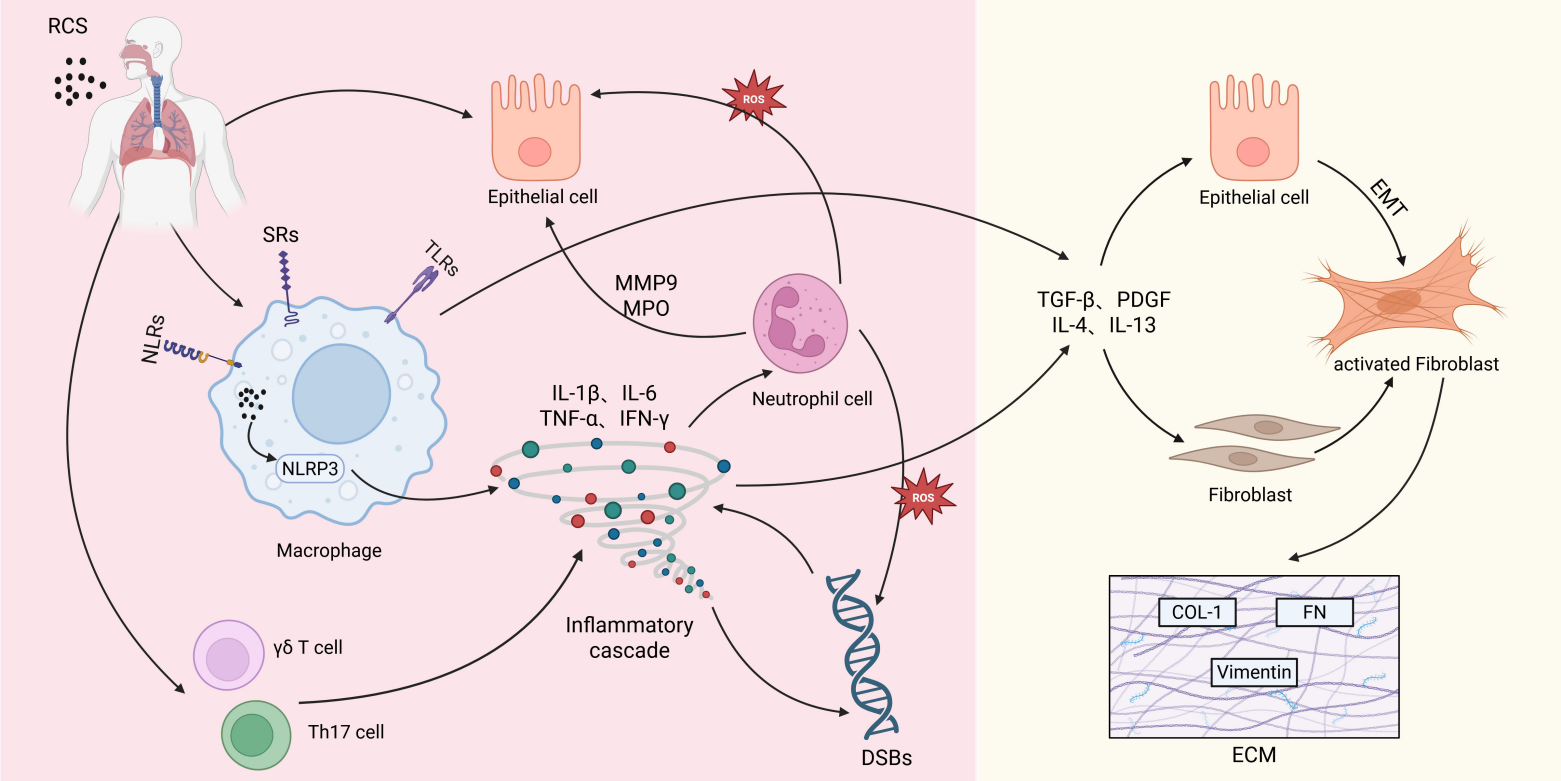
This mechanistic diagram illustrates the key pathological processes in silicosis pathogenesis. Alveolar macrophages recognize and phagocytose respirable crystalline silica (RCS), activating the NLRP3 inflammasome and triggering a robust inflammatory cascade. Concurrently, these inflammatory cytokines recruit immune cells such as neutrophils, resulting in excessive ROS production and aberrant elevation of oxidative stress. Within the persistently inflammatory microenvironment, activated immune cells secrete pro-fibrotic factors including TGF-β, PDGF, IL-4, and IL-13, which drive epithelial-mesenchymal transition (EMT) and fibroblast-to-activated fibroblast transition. These processes lead to a marked upregulation of extracellular matrix (ECM) component synthesis, ultimately causing irreversible pulmonary fibrosis. (NLRs, nucleotide-binding oligomerization domain (NOD)-like receptors; SRs, scavenger receptors; TLRs, Toll-like receptors; NLRP3, NOD-like receptor family pyrin domain containing 3; IL-1β, interleukin-1 beta; IL-6, interleukin-6; TNF-α, tumor necrosis factor alpha; IFN-γ, Interferon-gamma; MMP9, matrix metalloproteinase-9; MPO, myeloperoxidase; DSBs, double-strand breaks; TGF-β, transforming growth factor-beta; PDGF, platelet-derived growth factor; IL-4, interleukin-4; IL-13, interleukin-13; COL-I, type I collagen; FN, fibronectin).

RCS-adsorbed Fe^2+^ catalyzes hydroxyl radical (·OH) generation via Fenton reactions, triggering dysregulated intracellular redox homeostasis characterized by ROS overaccumulation (24). Notably, recent studies highlight ferroptosis, a distinct iron-dependent form of regulated cell death, as a critical mechanism in silicotic fibrosis. RCS-induced ROS overload and iron dysmetabolism drive lethal lipid peroxidation, which culminates in ferroptosis primarily via dysregulation of the key antioxidant enzyme glutathione peroxidase 4 (GPX4) (25, 26). Beyond ferroptosis, excessive ROS also induces mitochondrial membrane potential depolarization and cytochrome C release, activating the caspase-3-dependent apoptotic pathway (27). Additionally, RCS exhibit nuclear membrane permeability, mediating DNA double-strand breaks (DSBs) through direct physical disruption and NLRP3 inflammasome-dependent mechanisms. This genomic damage activates the ATM-ATR-LPA signaling cascade, establishing a vicious cycle of “DNA damage–inflammation” that promotes pulmonary fibrosis and exacerbates genomic instability (28, 29). The key pathological processes involved in silicosis pathogenesis are illustrated in the mechanistic diagram (**Figure 1**).

## 3 Limitations of current therapeutic approaches

### 3.1 Deficiencies in early diagnostic techniques

In current clinical practice, conventional radiographic imaging techniques demonstrate low sensitivity and poor specificity in identifying early-stage silicotic fibrotic lesions, frequently resulting in diagnostic confusion with other pulmonary conditions. Although high-resolution computed tomography (HRCT) offers superior detection capability for micronodular lesions, its restricted accessibility in primary healthcare facilities, compounded by patients’ financial constraints and radiation concerns, results in delayed diagnosis. Such diagnostic latency leads to late-stage identification in most patients, when pulmonary fibrosis has already progressed to moderate or advanced stages, significantly diminishing the window for effective intervention and compromising overall therapeutic outcomes.

### 3.2 Suboptimal efficacy in advanced disease management

The hallmark of silicosis lies in the progressive and irreversible nature of pulmonary fibrosis, posing a persistent therapeutic challenge. The recent approval by the U.S. Food and Drug Administration (FDA) of nintedanib (30) and jacrandilast (nerandomilast) (31) marks a pivotal advance, providing the first pharmacologic options specifically indicated to slow lung function decline in progressive silicosis. However, despite this progress, fundamental therapeutic limitations remain. Similar to traditional anti-fibrotic agents such as tetrandrine (32) and pirfenidone (33), the efficacy of these newer drugs is limited to attenuating disease progression without achieving regression of established fibrotic lesions. Furthermore, prolonged administration of these pharmacological agents is associated with significant adverse effects, including immunosuppression, hepatotoxicity, and gastrointestinal complications, which collectively compromise treatment adherence and clinical outcomes. Patients with advanced-stage (stage III) silicosis or concomitant respiratory failure exhibit a significantly reduced 5-year survival rate (10). As the definitive therapeutic modality for end-stage silicosis, lung transplantation can achieve a median survival duration of approximately 8 years post-transplantation. Nevertheless, multiple constraints severely limit its utilization, particularly the critical shortage of suitable donor organs, significant perioperative risk, and prohibitive costs (34). In the context of palliative management, although long-term oxygen therapy (LTOT) and non-invasive ventilation (NIV) provide symptomatic relief for dyspnea, these interventions are unable to reverse existing fibrotic lesions or reconstruct damaged alveolar structures.

### 3.3 Complexities in comorbidity management

Patients with silicosis demonstrate heightened susceptibility to Mycobacterium tuberculosis infection, attributable to compromised pulmonary architecture and systemic immune dysregulation (35). Silicosis-induced alveolar macrophage dysfunction compromises the phagocytic elimination of Mycobacterium tuberculosis, while the immunosuppressive pulmonary microenvironment fostered by chronic RCS exposure attenuates the therapeutic response to first-line antituberculosis regimens (36). Regarding cardiovascular complications, progressive pulmonary fibrosis contributes to its morbidity through chronic hypoxia-mediated mechanisms. The sustained hypoxic state induces pulmonary hypertension via synergistic acute vasoconstrictive responses and chronic vascular remodeling processes involving endothelial cell proliferation and extracellular matrix deposition. These pathological alterations progress irreversibly, culminating in structural reorganization of the pulmonary vasculature and subsequent cor pulmonale development. Concurrently, the chronic inflammatory microenvironment propagates pan-vascular endothelial dysfunction, accelerating atherosclerotic pathogenesis and elevating risks of coronary artery disease (37, 38). The current multidisciplinary therapeutic strategy, comprising LTOT, endothelin receptor antagonists, and anticoagulation regimens, demonstrates transient efficacy in improving hemodynamic parameters but is incapable of reversing existing pathological vascular remodeling and right ventricular hypertrophy. Critically, a subset of patients develops refractory right heart failure despite guideline-directed therapy, culminating in adverse clinical outcomes.

## 4 Mechanistic insights into stem cell therapy

### 4.1 Paracrine effects

Stem cells play a pivotal role in maintaining tissue microenvironment homeostasis through the paracrine secretion of diverse bioactive mediators that orchestrate intercellular signaling. Evidence indicates that these paracrine factors exert synergistic effects in attenuating localized inflammatory responses, promoting angiogenesis, and modulating fibrotic progression. This comprehensive modulation of tissue dynamics facilitates tissue regeneration and contributes to the functional recovery of compromised tissues (39). Regarding the mechanisms of inflammatory regulation, bone marrow-derived mesenchymal stem cells (BMSCs) secrete tumor necrosis factor-stimulated gene 6 (TSG-6), which potently inhibits RCS-induced NLRP3 inflammasome activation in macrophages. This TSG-6-mediated suppression markedly reduces IL-1β secretion, mitigating RCS-triggered acute pulmonary inflammation and exerting a protective effect on lung tissue (40). During disease progression, sustained inflammatory responses disrupt tissue repair processes, resulting in a pathological transition from acute inflammation to irreversible chronic fibrotic remodeling. A central pathogenic mechanism underlying pulmonary fibrosis involves the aberrant activation of fibroblasts into activated fibroblast. Recent studies demonstrate that exosomes derived from human umbilical cord mesenchymal stem cells (hucMSCs) transport microRNA let-7i-5p, which specifically inhibits the activation of TGF-β1/Smad3 signaling pathway. This molecular intervention effectively downregulates the expression of α-SMA, COL-I and FN, attenuates fibroblast activation, and reduces excessive ECM deposition, thereby attenuating the progression of pulmonary fibrosis (41).

Furthermore, the paracrine signaling of stem cells extends to the regulation of novel cell death pathways relevant to fibrotic progression. Emerging evidence highlights the potential of stem cells in regulating ferroptosis. Recent studies show that exosomes from menstrual blood−derived stem cells (MenSCs) deliver miR−let−7 to alveolar epithelial cells, where it targets and downregulates the transcription factor Sp3. This suppression reduces Sp3−mediated recruitment of histone deacetylase 2 (HDAC2), thereby relieving HDAC2−dependent repression of the Nrf2 antioxidant pathway. Subsequent Nrf2 activation enhances cellular defenses against lipid peroxidation and ultimately inhibits ferroptosis in experimental pulmonary fibrosis (42). While direct evidence from silicosis models is still needed, this mechanistic insight suggests that targeting ferroptosis via stem cell paracrine signaling could represent a promising therapeutic approach against silica-induced pulmonary fibrosis. Beyond this, the paracrine factors of stem cells also hold significant potential for mitigating vascular complications by promoting angiogenesis and inhibiting pathological vascular remodeling, positioning them as a dual-target therapy against both fibrosis and its cardiovascular sequelae (43).

### 4.2 Differentiation potential

The differentiation potential of stem cells refers to their capacity to differentiate into distinct cell lineages, classically categorized by hierarchical potency levels: totipotency, pluripotency, multipotency, and unipotency. The differentiation capacity is precisely regulated through an integrated multilayered control system encompassing: (1) core transcriptional regulatory networks, (2) dynamic epigenetic modifications, and (3) physicochemical microenvironmental cues (44). In recent years, stem cell therapy has demonstrated significant therapeutic potential for diverse pulmonary pathologies, with its distinctive regenerative mechanisms presenting innovative treatment strategies for silicosis management. Studies by Elga Bandeira et al. have demonstrated that localized tracheal administration of adipose-derived mesenchymal stromal cells (AD-MSCs) and their extracellular vesicles (EVs) effectively suppresses pulmonary expression of pro-inflammatory mediators IL-1β and TNF-α, ameliorates RCS-induced pulmonary inflammation, and significantly reduces collagen deposition and granuloma formation (45). Airway epithelial injury represents a critical pathogenic mechanism in silicosis progression. Recent studies demonstrate that human embryonic stem cell-derived MSC-like immune and matrix regulatory cells (hESC-IMRCs) can effectively reverse RCS-induced damage to human bronchial epithelial cells (HBECs) through activation of the Bmi1 signaling pathway. This cellular intervention restores epithelial proliferative capacity and differentiation potential while attenuating inflammatory cell infiltration and collagen deposition in murine pulmonary tissue, ultimately ameliorating fibrotic progression (46, 47).

### 4.3 Immunomodulation

There exists a sophisticated and intricate regulatory relationship between stem cells and the immune system. Research has demonstrated that stem cells exert immunomodulatory effects on both innate and adaptive immune responses via multiple mechanisms (48), including suppression of M1 macrophage polarization (49), promotion of Treg cell proliferation (50), and inhibition of Th17 cell activity (51). Additionally, stem cells maintain immune homeostasis through the secretion of anti-inflammatory cytokines such as IL-10 and TGF-β (52, 53) and exosome-mediated delivery of immunoregulatory miRNAs (54). Notably, the immunomodulatory strategies of Mesenchymal stem cells (MSCs), Airway basal stem cells (ABSCs), and Induced pluripotent stem cells (iPSCs) exhibit distinct emphases on specific immune axes, as summarized in **Table 1**. These immunomodulatory capabilities confer stem cells with extensive therapeutic applicability across diverse pathological conditions. In silicosis treatment, extracellular vesicles derived from human umbilical cord mesenchymal stem cells (hucMSC-EVs) have been demonstrated to modulate the circPWWP2A/miR-223-3p/NLRP3 signaling pathway. This regulatory mechanism effectively suppresses the secretion of pro-inflammatory cytokines, notably IL-1β and IL-18, while downregulating expression of fibrotic markers such as COL-I, COL-III, FN, and α-SMA. Collectively, these actions enable hucMSC-EVs to mitigate RCS-induced persistent pulmonary inflammation and aberrant collagen deposition in pulmonary tissues (55). miRNA sequencing profiling of hucMSC-EVs identified miR-148a-3p as the most abundant among differentially expressed miRNAs. miR-148a-3p expression was significantly suppressed in RCS-induced pulmonary fibrotic models, and therapeutic administration of hucMSC-EVs effectively restored its expression. Mechanistic studies further revealed that miR-148a-3p directly targets Hsp90b1 gene expression, consequently attenuating TGF-β1-mediated fibroblast activation, reducing α-SMA protein levels and collagen synthesis. These findings suggest that hucMSC-EVs may exert their anti-fibrotic effects through the miR-148a-3p/Hsp90b1 signaling pathway in pulmonary fibrosis (56).

**Table 1:** Comparison of advantages and limitations among MSCs, ABSCs and iPSCs.

Characteristics	Mesenchymal stem cells (MSCs)	Airway basal stem cells (ABSCs)	Induced pluripotent stem cells (iPSCs)
Cell Source	Allogeneic (derived from bone marrow, umbilical cord, etc.), widely available	Autologous (harvested from healthy airway tissue), requiring individualized collection	Autologous (derived from reprogrammed somatic cells), widely available yet inefficient in reprogramming
Immunogenicity	Low immunogenicity, suitable for allogeneic transplantation	Autologous cells, free from immune rejection	Autologous transplantation avoids rejection, but differentiated cells may retain residual immune risks
Core Mechanism	Immunoregulatory and reparative functions	Epithelial regeneration and tissue homeostasis	Multi-differentiation potential and epigenetic reprogramming
Immunomodulatory Profile	Broad-spectrum systemic regulation, primarily targeting macrophage polarization (M1/M2) and T-lymphocyte subsets (Treg/Th17) to suppress widespread inflammation and promote a pro-resolving microenvironment.	Site-specific regulation via epithelial−immune crosstalk and modulation of tissue−resident immune cells, primarily through structural repair and local paracrine signaling to dampen injury-site inflammation.	Enables precise macrophage reprogramming (e.g., M2 polarization) and generation of patient−specific regulatory immune cells (e.g., Tregs), offering mechanistically targeted and personalized therapeutic strategies.
Delivery Method	Intravenous or airway administration	Site-specific airway administration	Site-directed transplantation to target tissues
Persistence of Therapeutic Benefit	Demonstrates rapid efficacy but requires repeated administration	Sustained regeneration after one-time intervention	Potential long-term efficacy, but stability requires further validation
Treatment Safety	Systemic administration carries off-target risks.	Autologous cells demonstrate fewer local side effects	High tumorigenic risk necessitates enhanced QC measures
Target Population	Broad-spectrum applicability	Requires residual healthy lung tissue for collection	Theoretically applicable to all patients
Preparation Difficulty and Cost	Standardized production with lower costs	Individualized production necessitates extended durations and elevated expenses	Reprogramming and differentiation processes are technically demanding and cost-prohibitive
Regenerative capacity	Capable of multi-organ modulation	Accurate therapeutic intervention for alveolar lesions	Suitable for personalized therapy and disease modeling.
Clinical Trial Status	Early-phase clinical trials ongoing (e.g., NCT01239862). Safety established; Efficacy under investigation.	No clinical trials in silicosis. Mainly studied in preclinical models for epithelial repair.	No clinical trials in silicosis. Used primarily for disease modeling and drug screening.
Ethical Controversies	Minimal controversy	Minimal controversy	Involves embryo-like states, raising significant controversies
References	(40)、 (55)、 (61)	(46)、 (62)	(65)、 (66)

**Figure 2:**
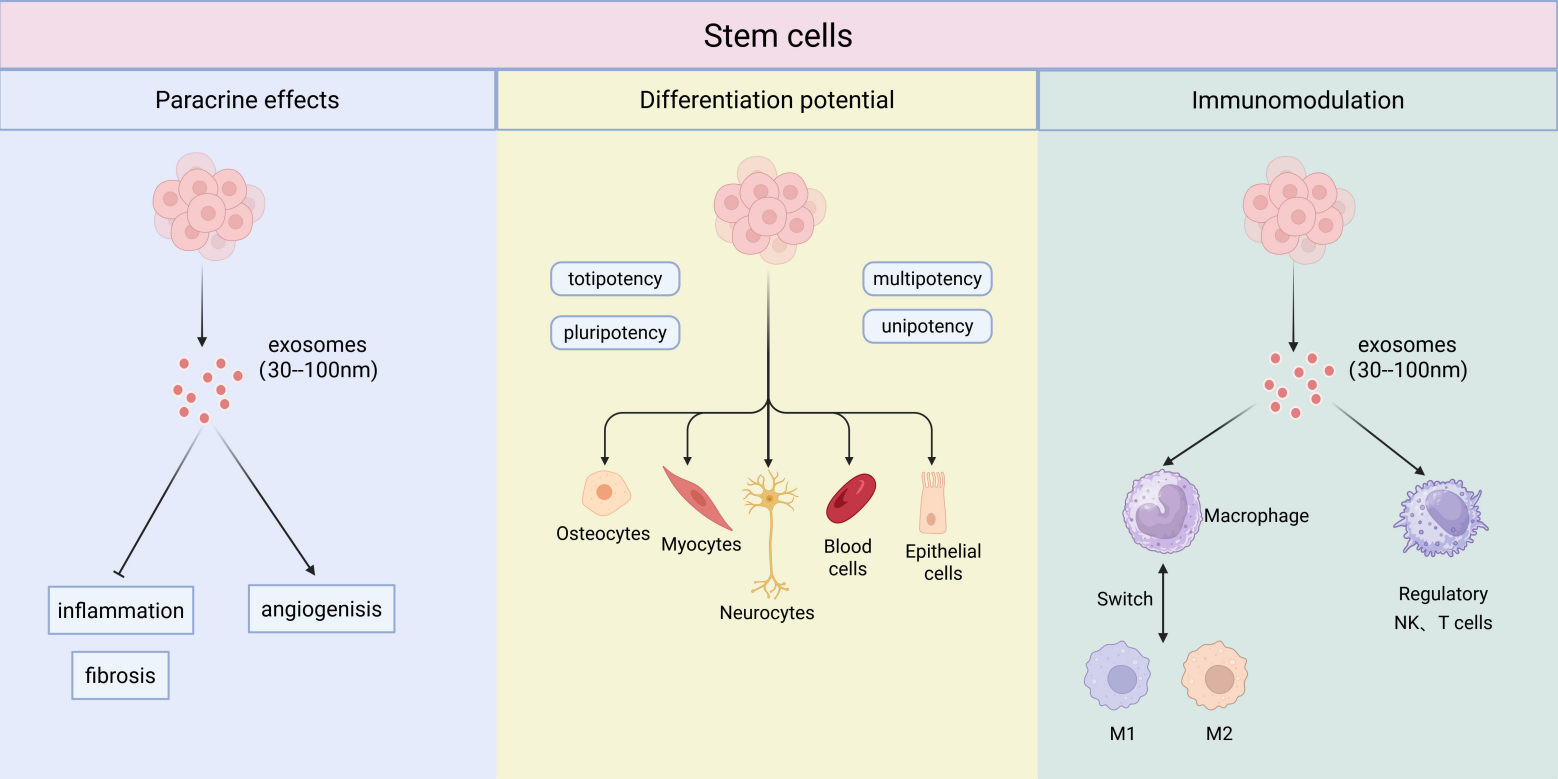
The multifaceted mechanisms of stem cell therapy. Stem cells exert their therapeutic effects through three primary mechanisms: paracrine effects, differentiation potential, and immunomodulation. The paracrine effects are largely mediated by the release of exosomes (30–100 nm), which influence processes such as inflammation, angiogenesis, and fibrosis. Their differentiation potential ranges from totipotency and pluripotency to multipotency and unipotency, giving rise to various cell lineages. A key immunomodulatory function is the switching of macrophage polarization from a pro-inflammatory M1 phenotype to an anti-inflammatory, regenerative M2 phenotype. Furthermore, NK, and T cells also play an important role in establishing the regulatory immune environment.

Beyond mitigating fibrosis-associated inflammation, the immunomodulatory capacity of MSCs holds significant promise for addressing infectious complications in silicosis. Recent advances demonstrate that MSCs can be engineered to enhance this antimicrobial potential. It has been shown that BMSCs genetically modified to express the antimicrobial peptide PK34 acquire direct bactericidal activity against Mycobacterium tuberculosis. Furthermore, these engineered cells synergistically modulate the immune response by increasing anti-inflammatory cytokines and protecting host immune cells, thereby reducing both bacterial burden and infection-related pathology (57). This evidence underscores the potential of leveraging and enhancing MSC-based immunomodulation to address critical comorbidities in silicosis. The multifaceted mechanisms of stem cell therapy are summarized in **Figure 2**.

## 5 Selection of optimal stem cells sources

Stem cells, representing a cornerstone of regenerative medicine, have demonstrated extensive therapeutic potential across diverse diseases owing to their self-renewal capacity and multilineage differentiation capacity. Based on ontogenic origin, stem cells are primarily categorized into embryonic stem cells (ESCs) derived from the inner cell mass of blastocysts, tissue-resident adult stem cells, and induced pluripotent stem cells (iPSCs) generated through somatic cell reprogramming (58). In recent years, the breakthroughs in gene editing technologies, three-dimensional bioprinting systems, and organoid culture platforms have significantly advanced stem cell-based therapeutics, with demonstrated efficacy in tissue regeneration, drug development, and age-related disease modulation.

### 5.1 Mesenchymal stem cells

Mesenchymal stem cells (MSCs), a class of multipotent stromal cells of mesodermal origin, demonstrate robust self-renewal capacity and multipotent differentiation properties. These cells are ubiquitously distributed in various tissues including bone marrow stroma, adipose vasculature, umbilical cord Wharton’s jelly, and placental decidua. Their unique biological properties such as abundant sources, easy accessibility, and low immunogenicity, position MSCs as particularly promising therapeutic candidates for silicosis treatment (59). Studies have demonstrated that BMSCs modulate macrophage NLRP3 inflammasome activation through TSG-6 secretion, significantly attenuating RCS-induced acute pulmonary inflammation (40). Concurrently, BMSCs suppress the production of pro-inflammatory cytokines and reverse the aberrant expression of fibrotic markers through inhibition of the TGF-β/Smad signaling pathway (60).

Notably, there is a growing research focus on cell-free therapeutic strategies based on mesenchymal stem cells, particularly their derived extracellular vesicles. Substantial evidence indicates that the therapeutic effects of MSCs are predominantly mediated by paracrine mechanisms, with exosomes serving as critical carriers of bioactive components. Recent studies have demonstrated that, in a silicosis mouse model, intravenous administration of human umbilical cord MSC-derived extracellular vesicles (hucMSC-EVs) exhibited comparable efficacy to their parental cells in reducing lung collagen deposition and downregulating key fibrotic marker proteins. Mechanistically, hucMSC-EVs have been shown to mitigate macrophage-driven inflammatory responses and suppress fibroblast activation and proliferation through the circPWWP2A/miR-223-3p/NLRP3 signaling pathway (55). Furthermore, emerging research indicates that MSC-EVs can deliver miR-99a-5p to inhibit FGFR3 and its downstream MAPK signaling, thereby effectively blocking the pathological activation of fibroblasts. Importantly, exosomes derived from MSCs overexpressing miR-99a-5p demonstrate significantly enhanced anti-fibrotic effects, suggesting the potential for engineering EVs to optimize therapeutic outcomes (61). In summary, accumulating evidence indicates that MSC-EVs can efficiently deliver the core therapeutic components of MSCs. While achieving comparable anti-fibrotic efficacy, they hold the promise of mitigating potential risks associated with whole-cell therapies, such as issues related to cell viability, aberrant differentiation, and pulmonary vascular occlusion, thereby highlighting their unique advantages as a next-generation therapeutic strategy.

### 5.2 Airway basal stem cells

Airway basal stem cells (ABSCs) serve as the principal effector cells orchestrating respiratory epithelial repair and regeneration, exhibiting considerable therapeutic potential for chronic pulmonary diseases. The maintenance of respiratory mucosal epithelial homeostasis is fundamentally dependent on tissue-resident stem cell populations, including ABSCs, secretory progenitor cells, and alveolar type II epithelial stem cells. ABSCs occupy a central position in pulmonary epithelial homeostasis, owing to their capacity for self-renewal and multipotent differentiation, which underpins their critical functions in mucociliary clearance mechanisms and epithelial barrier restoration (62). During silicosis pathogenesis, chronic exposure to RCS triggers sustained inflammatory responses and oxidative stress, resulting in recurrent airway epithelial injury and ultimately leading to the progressive depletion of ABSCs. As the disease progresses, the consequent decline in regenerative capacity drives pathological airway remodeling and irreversible pulmonary interstitial fibrosis. Yang et al. discovered significant downregulation of Bmi1, a crucial Polycomb group transcriptional regulator, in experimental silicosis murine models. *In vitro* studies demonstrated that Bmi1 overexpression not only promoted proliferative capacity, enhanced differentiation potential, and restored epithelial regenerative function in RCS-exposed HBECs, but also substantially ameliorated senescence-related cellular phenotypes. These findings suggest that Bmi1 may serve as a critical transcriptional regulator governing ABSCs self-renewal dynamics, proliferative activity, and differentiation programs during silicosis progression, providing evidence positioning Bmi1 as a promising molecular target for developing stem cell-based therapeutic interventions against silicosis (46).

### 5.3 Induced pluripotent stem cells

Induced pluripotent stem cells (iPSCs), recognized as a major breakthrough in regenerative medicine, are generated through transcription factor-mediated reprogramming of differentiated somatic cells to acquire embryonic stem cell-like pluripotency (63). This groundbreaking technology was first established in murine fibroblasts by Yamanaka and colleagues in 2006 (64), with subsequent successful implementation in human somatic cell reprogramming reported by the same research group in 2007 (63). IPSCs exhibit essentially unlimited self-renewal capacity and multilineage differentiation potential, enabling their derivation into diverse functional cell lineages such as cardiomyocytes, neurons, and alveolar epithelial cells. Importantly, iPSC technology overcomes the ethical constraints inherent to ESCs research while maintaining comparable developmental plasticity. Currently, iPSCs have emerged as indispensable research tools with broad applications studying human developmental biology, establishing patient-specific disease models, facilitating high-throughput drug discovery, and evaluating compound toxicity. Furthermore, iPSCs show significant promise for clinical translation in regenerative medicine through cell replacement therapies (65). Beyond their established utility in disease modeling and drug screening, a particularly promising and clinically relevant derivative of iPSCs is their secreted exosomes (iPSC-Exos). In pulmonary fibrosis research, iPSC-Exos have demonstrated significant therapeutic efficacy by attenuating M2 macrophage polarization and reducing ECM deposition through selective modulation of the miR-302a-3p/TET1 signaling pathway, consequently mitigating fibrotic progression (66). However, despite these compelling advances in generic fibrosis models, the direct application and efficacy of iPSC-Exos in silicosis-associated pulmonary fibrosis remain experimentally unexplored, representing both a critical knowledge gap and a promising direction for future investigation.

## 6 Challenges in clinical translation

### 6.1 Technical hurdles

The implementation of Good Manufacturing Practice (GMP)-compliant production systems for stem cell therapeutics remains hindered by multiple unresolved technical and biological challenges. A critical barrier lies in the incomplete translation from research-grade to clinical-grade manufacturing, with persistent technical bottlenecks in standardizing critical culture parameters during somatic cell reprogramming, directed differentiation, and large-scale expansion. Furthermore, biological heterogeneity among donors leads to substantial batch-to-batch variability in epigenetic characteristics, secretome profiles, and functional potency of the final product. During *in vitro* scale-up, progressive stemness attenuation during serial passaging and insufficient standardization of cryopreservation-recovery workflows compromise product consistency (67). To address these systemic bottlenecks, recent guidelines from the International Society for Cell and Gene Therapy (ISCT) advocate for a comprehensive, function-centric standardization framework. Key actionable measures encompass the strict control of culture conditions, including restricting expansion to early passages to maintain cell potency, and the adoption of defined culture media to reduce batch-to-batch variability (68). Crucially, quality assurance must extend beyond minimal phenotypic criteria to incorporate relevant functional potency assays that align with the intended therapeutic mechanism. The implementation of Process Analytical Technology (PAT) within closed, automated bioreactor systems is also emphasized to enable real-time monitoring and dynamic control of critical process parameters, thereby enhancing manufacturing consistency and compliance (69). These strategies form the foundational basis for establishing clinically compliant, reproducible, and scalable manufacturing platforms for stem cell-based therapeutics.

The clinical efficacy of stem cell therapy primarily depends on the post-transplantation viability of administered cells and their targeted homing efficiency. Studies demonstrate that fibrotic microenvironments substantially compromise transplanted stem cell survival, with quantitative analyses showing a rapid decline in pulmonary tissue homing efficiency within 72 hours post-transplantation, ultimately limiting therapeutic outcomes (70). In experimental silicosis models, the pathognomonic oxidative stress environment, aberrant collagen deposition, and elevation of profibrotic mediators collectively exert suppressive effects on transplanted stem cell viability and functional potency. Although stem cells exhibit anti-fibrotic potential via paracrine mechanisms, their engraftment efficiency in pulmonary tissue remains suboptimal, and conclusive evidence of functional differentiation into lung-specific cell lineages remains elusive. To overcome the critical barrier of suboptimal homing, a multifaceted strategy focusing on both the cells and the delivery system is essential. The most direct approach is optimizing the administration route. For a focal lung disease such as silicosis, local airway administration offers a direct pathway to the target tissue. Theoretical and preclinical evidence strongly supports that this approach enhances pulmonary cell retention and local therapeutic bioavailability compared to systemic intravenous infusion. Concurrently, enhancing the intrinsic homing capacity of MSCs through bioengineering represents a key research frontier. This includes genetic modification such as the overexpression of CXCR4/CXCR7 and surface engineering of adhesion molecules to strengthen chemotactic and adhesive responses, as well as external guidance systems like magnetic targeting to physically direct cells to the lesion site. Complementing these cellular approaches, modifying the lung microenvironment via localized release of chemokines like SDF-1 can create a potent “homing beacon” to recruit circulating or locally delivered MSCs (71).

Therefore, developing integrated strategies that combine standardized manufacturing of potent cell products with innovative bioengineering for targeted delivery and microenvironmental priming represents a pivotal research priority for regenerative medicine in pulmonary fibrosis (72).

### 6.2 Safety concerns

The principal barrier to clinical implementation of stem cell therapies lies in persistent safety considerations, including teratoma formation from residual undifferentiated cells, host immune responses, and uncontrolled differentiation. Current research demonstrates that residual undifferentiated iPSCs retain substantial oncogenic potential, principally stemming from reprogramming-induced genomic instability and progressive epigenetic dysregulation during prolonged culture expansion. From an immunological standpoint, the tumorigenic potential of transplanted cells exhibits an intricate dependence on their immunogenic properties. while vigorous immune surveillance can effectively eliminate malignant transformation, it simultaneously enhances the likelihood of graft rejection. Reciprocally, diminished immunogenicity promotes successful engraftment while potentially compromising tumor surveillance mechanisms, thereby elevating oncogenic risk (73). Addressing this critical safety concern, Yagyu et al. made a pivotal contribution to cellular safety engineering by developing a Caspase-9-based suicide gene system that reliably eliminates residual teratoma-forming iPSCs, thereby overcoming a fundamental translational barrier in regenerative medicine (74).

### 6.3 Ethical and regulatory controversies

While stem cell research demonstrates remarkable potential for advancing scientific knowledge and developing novel therapeutics, it simultaneously engenders complex ethical and policy considerations. These controversies primarily center on the ontological and moral status of human embryos, biosafety and technological risks associated with stem cell applications, and sociopolitical concerns regarding equitable access and distributive justice in emerging therapies. As a unique cell type possessing pluripotent differentiation capabilities, ESCs require the destruction of early-stage embryos for research purposes, which has sparked profound philosophical and ethical debates across academic and societal domains. While the development of iPSCs through somatic cell reprogramming offers an alternative that mitigates these ethical dilemmas, this approach introduces its own set of challenges including the potential risks associated with genetic manipulation and concerns over the commercialization and unethical exploitation of stem cell technologies. The current academic consensus emphasizes that stem cell research must adhere to principles of scientific rigor, stringent informed consent, and equitable distribution. This approach requires establishing regulatory systems that harmonize technological innovation with ethical accountability, achieved through interdisciplinary collaboration and robust legal frameworks to ensure technological advancements remain aligned with established ethical norms (75–77).

## 7 Next-generation perspectives and innovative strategies

### 7.1 Gene-editing enhanced stem cell

Recent years have witnessed the ascendance of combinatorial approaches merging stem cell therapy with gene editing technologies, creating novel therapeutic avenues that are redefining the landscape of regenerative medicine. The CRISPR/Cas9 system has emerged as a transformative genome-editing platform in stem cell research due to its high efficiency and precision. This molecular machinery operates through a sequence-specific targeting mechanism, wherein the Cas9 endonuclease is precisely guided to predetermined genomic loci by complementary single-guide RNA (sgRNA). The ribonucleoprotein complex recognizes and cleaves target DNA sequences immediately upstream of protospacer adjacent motif (PAM) sites, generating site-specific double-strand breaks. These induced DNA lesions subsequently engage the cell’s intrinsic repair pathways, predominantly activating either the error-prone non-homologous end joining (NHEJ) pathway for gene knockout or the high-fidelity homology-directed repair (HDR) pathway for precise gene correction, thereby enabling diverse genomic modifications (78). The application of this genome-editing platform in stem cell therapeutics has been successfully employed for precise genetic modifications in pluripotent stem cells (79–81), hematopoietic stem cells (82, 83), and adult stem cells (84–86). Furthermore, its therapeutic efficacy has been demonstrated across multiple disease models, including retinal degenerative disorders (87, 88), neural injury repair (89, 90), and osteochondral regeneration (91). Building upon this established technological foundation, a compelling next-generation strategy for silicosis involves the deliberate engineering of therapeutic stem cells to directly counteract its specific pathogenic drivers. Rational target selection is paramount and can be guided by key insights from silicosis pathophysiology. For instance, CRISPR-mediated overexpression of potent anti-inflammatory mediators such as TSG-6 in MSCs could be employed to amplify their capacity to suppress the NLRP3 inflammasome, a key driver of silicotic inflammation (40). Similarly, engineering airway basal stem cells (ABSCs) to overexpress pro-regenerative transcription factors like Bmi1 presents a promising approach to enhance epithelial repair in the fibrotic niche, given the essential role of Bmi1 in stem cell maintenance and its depletion in fibrotic lungs (46). The systematic creation and evaluation of such “designer” stem cells represent a nascent but profoundly innovative frontier in silicosis therapeutics, aiming to produce optimized cellular agents specifically engineered for the diseased lung microenvironment.

However, the clinical translation of CRISPR-based therapeutics continues to encounter significant challenges, including off-target editing events, suboptimal delivery efficiency, and host immune responses. To advance this specific application in regenerative medicine for silicosis, future investigations should systematically address these technical limitations while comprehensively evaluating the long-term biosafety profiles of such genetically enhanced stem cells through standardized preclinical assessments in relevant models of silica-induced lung injury (92).

### 7.2 Stem cell-driven nano-delivery systems

The integration of nanotechnology with stem cell regenerative medicine has emerged as a cutting-edge interdisciplinary research frontier. Nanotechnology demonstrates significant potential in stem cell-targeted delivery, high-resolution sorting, directed fate modulation, and biomimetic tissue regeneration through its unique physicochemical properties. In differentiation control, biomaterials such as collagen-based nanofibrous scaffolds (93) and graphene oxide nanoparticles (94) can mimic the physicochemical characteristics of stem cell microenvironments, enabling precise regulation of stem cell differentiation through activation of specific molecular signaling pathways. Contemporary research advancements reveal that biocompatible nanoparticles not only achieve high-precision stem cell isolation but also permit non-invasive, real-time tracking of transplanted stem cells, providing molecular-level observation tools to investigate stem cell migration patterns and their functional interaction with target tissues (95, 96). Furthermore, biomimetic membrane-modified functionalized nanoparticles can effectively improve stem cell adhesion at target sites and enhance homing efficiency (97). However, the clinical translation of nanocarrier-based stem cell modulation remains constrained by unresolved biosafety concerns. Critical issues requiring further investigation include the cytotoxicity mechanisms of nanomaterials, the potential interference with stem cell differentiation pathways, and the intracellular metabolic processing and clearance dynamics.

### 7.3 Stem cell-derived organoid technologies

Organoid technology represents a transformative breakthrough in regenerative medicine, with its origins tracing back to the groundbreaking work of Hans Clever’s team in 2009. Their pioneering achievement in generating intestinal organoids with characteristic crypt-villus structures from intestinal stem cells not only marked the advent of *in vitro* organ reconstruction technology but also established a crucial foundation for subsequent advancements in the field (98). Building upon this technological platform, the research team led by Seok-Ho Hong achieved successful differentiation of human pluripotent stem cells (hPSCs) into alveolar organoid (AOs) models. These sophisticated models comprise alveolar stem cells, alveolar epithelial cells (AEC1/AEC2), and mesenchymal cells, demonstrating high pathological fidelity in modeling critical aspects of pulmonary fibrosis, including alveolar epithelial injury, epithelial-mesenchymal transition, and abnormal collagen deposition (99). Expanding on this work, the research team generated multicellular alveolar organoids containing functional macrophages derived from hPSCs. This was achieved through a precisely controlled, stepwise differentiation protocol that recapitulates developmental signaling pathways in a temporally regulated manner. The establishment of this advanced model system provided the first direct *in vitro* evidence of macrophages’ pivotal roles in modulating inflammation and promoting collagen clearance (100). As a distinct subtype of pulmonary fibrosis, silicosis shares core pathological hallmarks including alveolar epithelial injury, dysregulated inflammation, and aberrant collagen deposition, which are effectively modeled in established alveolar organoid systems (101, 102). Building upon this foundation, the development and rigorous validation of a silicosis-specific alveolar organoid model represent a critical next step. Such a model would ideally be established by exposing AOs to RCS, with the explicit goal of recapitulating disease-defining features such as the sustained inflammation-fibrosis cascade and the complex architecture of silicotic nodules. Future research directions should focus on enhancing organoid complexity through the incorporation of critical microenvironmental components, and integrating with advanced technologies such as CRISPR-based gene editing and single-cell RNA sequencing, thereby providing a powerful platform for mechanistic investigation of silicosis pathogenesis and preclinical evaluation of targeted therapeutic strategies.

## 8 Discussion

In summary, silicosis, an interstitial lung disease characterized by irreversible fibrosis, currently only has therapeutic options that can delay disease progression. Therefore, there is an urgent need for novel therapeutic strategies that target the pathological root causes of this disease. Despite substantial progress in elucidating its molecular pathogenesis, current therapeutic modalities demonstrate limited efficacy in halting disease progression, offering merely palliative attenuation of fibrotic processes. In recent years, stem cell-based regenerative medicine has emerged as a promising therapeutic strategy for silicosis management. Stem cell therapy simultaneously targets inflammatory responses and fibrotic processes through multimodal therapeutic mechanisms such as paracrine effects and immunomodulation, which has shifted the treatment paradigm from single-target interventions to multi-mechanism synergistic approaches. However, clinical translation of stem cell therapy still faces critical challenges, including standardization of production processes, long-term safety concerns, and ethical controversies.

Future research should focus on integrating innovative strategies, including gene editing technologies, intelligent nano-delivery systems, and organoid models, to enhance therapeutic efficacy. A pivotal and rational research direction involves the systematic development of combination therapies. This paradigm could explore synergistic regimens that co-administer stem cells with approved anti-fibrotic agents) to concurrently target multiple nodes of the fibrotic cascade. Concurrently, pre-engineering stem cells via CRISPR/Cas9 to overexpress specific therapeutic factors represents a logical strategy to augment their intrinsic reparative capacity. Furthermore, a comprehensive therapeutic framework for silicosis should also explicitly encompass strategies to manage its major comorbidities, such as tuberculosis and pulmonary hypertension. This may involve the design of stem cells engineered to serve as targeted delivery vehicles for adjunctive therapeutics. Through interdisciplinary collaboration, stem cell therapy holds significant potential to provide breakthrough treatments for refractory lung diseases such as silicosis, ultimately improving both clinical outcomes and quality of life for affected patients.

## Glossary

RCS: respirable crystalline silica
MSC: mesenchymal stem cells
ABSCs: airway basal stem cells
iPSCs: induced pluripotent stem cells
WHO: World Health Organization
PAMPs: pathogen-associated molecular patterns
DAMPs: damage-associated molecular patterns
PRRs: pattern recognition receptors
SRs: scavenger receptors
TLRs: Toll-like receptors
NLRs: nucleotide-binding oligomerization domain (NOD)-like receptors
ROS: reactive oxygen species
NLRP3: NOD-like receptor family pyrin domain containing 3
MPO: myeloperoxidase
MMP-9: matrix metalloproteinase-9
RNS: reactive nitrogen species
NOX: Nicotinamide Adenine Dinucleotide Phosphate (NADPH) oxidase
O₂⁻: superoxide anions
H₂O₂: hydrogen peroxide
MMP-12: matrix metalloproteinase-12
TGF-β1: transforming growth factor-β1
PDGF: platelet-derived growth factor
ECM: extracellular matrix
COL-I: type I collagen
FN: fibronectin
MMP-1: matrix metalloproteinase-1
EMT: epithelial-mesenchymal transition
•OH: hydroxyl radical
DSBs: DNA double-strand breaks
IL-1β: interleukin-1 beta
IL-6: interleukin-6
TNF-α: tumor necrosis factor alpha
IFN-γ: Interferon-gamma
IL-4: interleukin-4
IL-13: interleukin-13
HRCT: high-resolution computed tomography
LTOT: long-term oxygen therapy
NIV: non-invasive ventilation
BMSCs: bone marrow-derived mesenchymal stem cells
TSG-6: tumor necrosis factor-stimulated gene 6
hucMSCs: human umbilical cord mesenchymal stem cells
AD-MSCs: adipose-derived mesenchymal stromal cells
EVs: extracellular vesicles
hESC-IMRCs: human embryonic stem cell-derived MSC-like immune and matrix regulatory cells
HBECs: human bronchial epithelial cells
hucMSC-EVs: extracellular vesicles derived from human umbilical cord mesenchymal stem cells
ESCs: embryonic stem cells
iPSC-Exos: exosomes derived from iPSCs
GMP: Good Manufacturing Practice
ISCT: International Society for Cellular Therapy
PAT: process analytical technology
sgRNA: single-guide RNA
PAM: protospacer adjacent motif
NHEJ: error-prone non-homologous end joining
HDR: homology-directed repair
hPSCs: human pluripotent stem cells
AOs: alveolar organoid
GPX4: glutathione peroxidase 4
MenSCs: menstrual blood-derived stem cells
HDAC2: histone deacetylase 2
